# Constructing an artificial intelligence-assisted system for the assessment of gastroesophageal valve function based on the hill classification (with video)

**DOI:** 10.1186/s12911-025-02973-1

**Published:** 2025-03-24

**Authors:** Jian Chen, Ganhong Wang, Kaijian Xia, Zhenni Wang, Luojie Liu, Xiaodan Xu

**Affiliations:** 1 Department of Gastroenterology, Changshu Hospital Affiliated to Soochow University, No. 1 Shuyuan Street, Suzhou, Jiangsu 215500 China; 2 Department of Gastroenterology, Changshu Hospital Affiliated to Nanjing University of Chinese Medicine, Suzhou, Jiangsu 215500 China; 3 Department of Information Engineering, Changshu Key Laboratory of Medical Artificial Intelligence and Big Data, Changshu City, Jiangsu Province 215500 China

**Keywords:** Gastroesophageal flap valve, Deep learning, Convolutional neural networks, Hill classification, Gastroscopy

## Abstract

**Objective:**

In the functional assessment of the esophagogastric junction (EGJ), the endoscopic Hill classification plays a pivotal role in classifying the morphology of the gastroesophageal flap valve (GEFV). This study aims to develop an artificial intelligence model for Hill classification to assist endoscopists in diagnosis, covering the entire process from model development, testing, interpretability analysis, to multi-terminal deployment.

**Method:**

The study collected four datasets, comprising a total of 1143 GEFV images and 17 gastroscopic videos, covering Hill grades I, II, III, and IV. The images were preprocessed and enhanced, followed by transfer learning using a pretrained model based on CNN and Transformer architectures. The model training utilized a cross-entropy loss function, combined with the Adam optimizer, and implemented a learning rate scheduling strategy. When assessing model performance, metrics such as accuracy, precision, recall, and F1 score were considered, and the diagnostic accuracy of the AI model was compared with that of endoscopists using McNemar’s test, with a *p*-value < 0.05 indicating statistical significance. To enhance model transparency, various interpretability analysis techniques were used, including t-SNE, Grad-CAM, and SHAP. Finally, the model was converted into ONNX format and deployed on multiple device terminals.

**Results:**

Compared through performance metrics, the EfficientNet-Hill model surpassed other CNN and Transformer models, achieving an accuracy of 83.32% on the external test set, slightly lower than senior endoscopists (86.51%) but higher than junior endoscopists (75.82%). McNemar’s test showed a significant difference in classification performance between the model and junior endoscopists (*p* < 0.05), but no significant difference between the model and senior endoscopists (*p* ≥ 0.05). Additionally, the model reached precision, recall, and F1 scores of 84.81%, 83.32%, and 83.95%, respectively. Despite its overall excellent performance, there were still misclassifications. Through interpretability analysis, key areas of model decision-making and reasons for misclassification were identified. Finally, the model achieved real-time automatic Hill classification at over 50fps on multiple platforms.

**Conclusion:**

By employing deep learning to construct the EfficientNet-Hill AI model, automated Hill classification of GEFV morphology was achieved, aiding endoscopists in improving diagnostic efficiency and accuracy in endoscopic grading, and facilitating the integration of Hill classification into routine endoscopic reports and GERD assessments.

## Introduction

The global incidence of Gastroesophageal Reflux Disease (GERD) ranges from 8% to 33%, marking it as a commonly encountered yet complex digestive system disorder [1, 2]. Research suggests that esophageal mucosal injury is primarily linked to anatomical or physiological defects at the Esophagogastric Junction (EGJ) [3]. In particular, the Gastroesophageal Flap Valve (GEFV), a key element of the EGJ, plays a significant role in the anti-reflux barrier [4]. The GEFV was first introduced by Tocornal et al. in 1968 [5], and its existence in the EGJ was later confirmed through autopsy by Thor and Hill in 1987 [6]. In 1996, Hill and others, based on the endoscopic characteristics of GEFV, proposed the Hill classification, defining Grades I–II as normal GEFV and Grades III–IV as abnormal [7].

Studies have shown that abnormal GEFV is closely associated with diseases such as GERD, Barrett’s esophagus, esophageal hiatal hernia, laryngopharyngeal reflux disease, dyspepsia, and esophageal variceal bleeding [8–11]. The Hill classification is a crucial metric for evaluating GERD before and after surgical or endoscopic treatments [12]. Due to its clarity, the Hill classification is favored by endoscopists. Scholars have advocated for its inclusion in routine endoscopic reports and GERD assessments [13]. However, physicians face a learning curve and challenges in assessment consistency when applying the Hill classification.

In recent years, the application of artificial intelligence in the field of digestive endoscopy has been increasing, particularly in aspects of quality control, diagnostic assistance, and decision support, bringing significant advancements to gastrointestinal endoscopy. Wang C and colleagues [14] utilized Convolutional Neural Networks (CNN) to develop a GERD grading model based on the Los Angeles classification criteria. Furthermore, H. Yen and others [15] introduced an innovative method combining deep learning with machine learning, significantly enhancing the accuracy of gastroesophageal reflux disease endoscopic classification, achieving a test accuracy of 92.5% ± 2.1%. Notably, in recent years, Transformer technology is rapidly gaining attention in the medical field, surpassing CNN and emerging as a new focus in this domain.

This study applies deep learning (DL) technology to assess the function of the Gastroesophageal Flap Valve (GEFV) and to develop a four-category deep learning model based on the Hill classification system, encompassing the entire process from model development, testing, interpretability analysis, to multi-terminal deployment. It employed two different deep learning architectures, CNN and Transformer, to construct seven distinct deep learning network models. In evaluating these models, a series of comprehensive performance metrics were used, including accuracy, precision, sensitivity, specificity, recall rate, F1 score, average precision, and AUC value. Additionally, the study compared the performance in classification accuracy between DL models and endoscopists of varying experience levels, offering insights for the practical application of deep learning in the medical field.

## Materials and methods

### Datasets

**Fig. 1:**
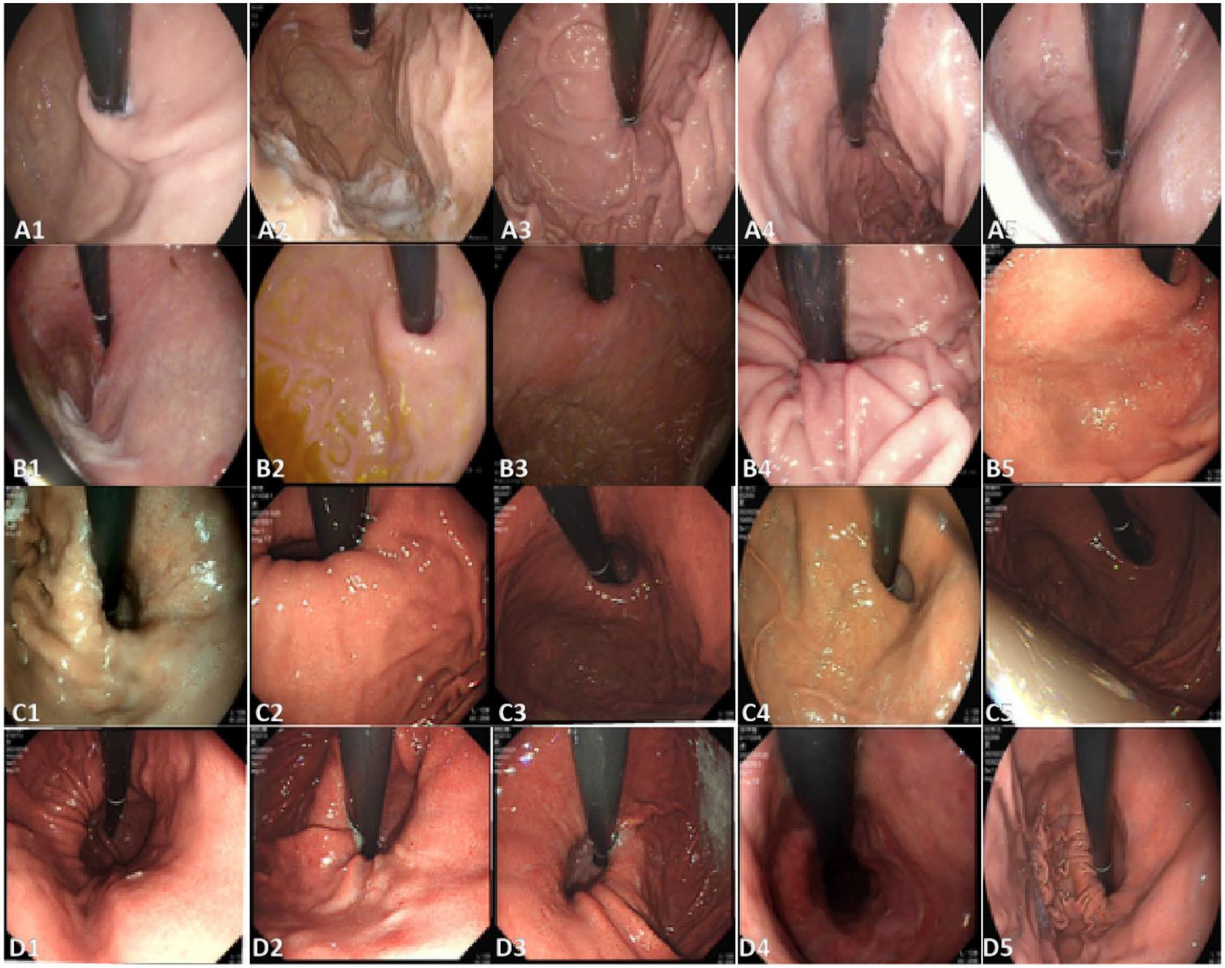
Relevant image examples from the dataset; **A**1–**A**5 represent Hill Grade I, **B**1–**B**5 denote Hill Grade II, **C**1–**C**5 illustrate Hill Grade III, and **D**1–**D**5 correspond to Hill Grade IV

**Fig. 2:**
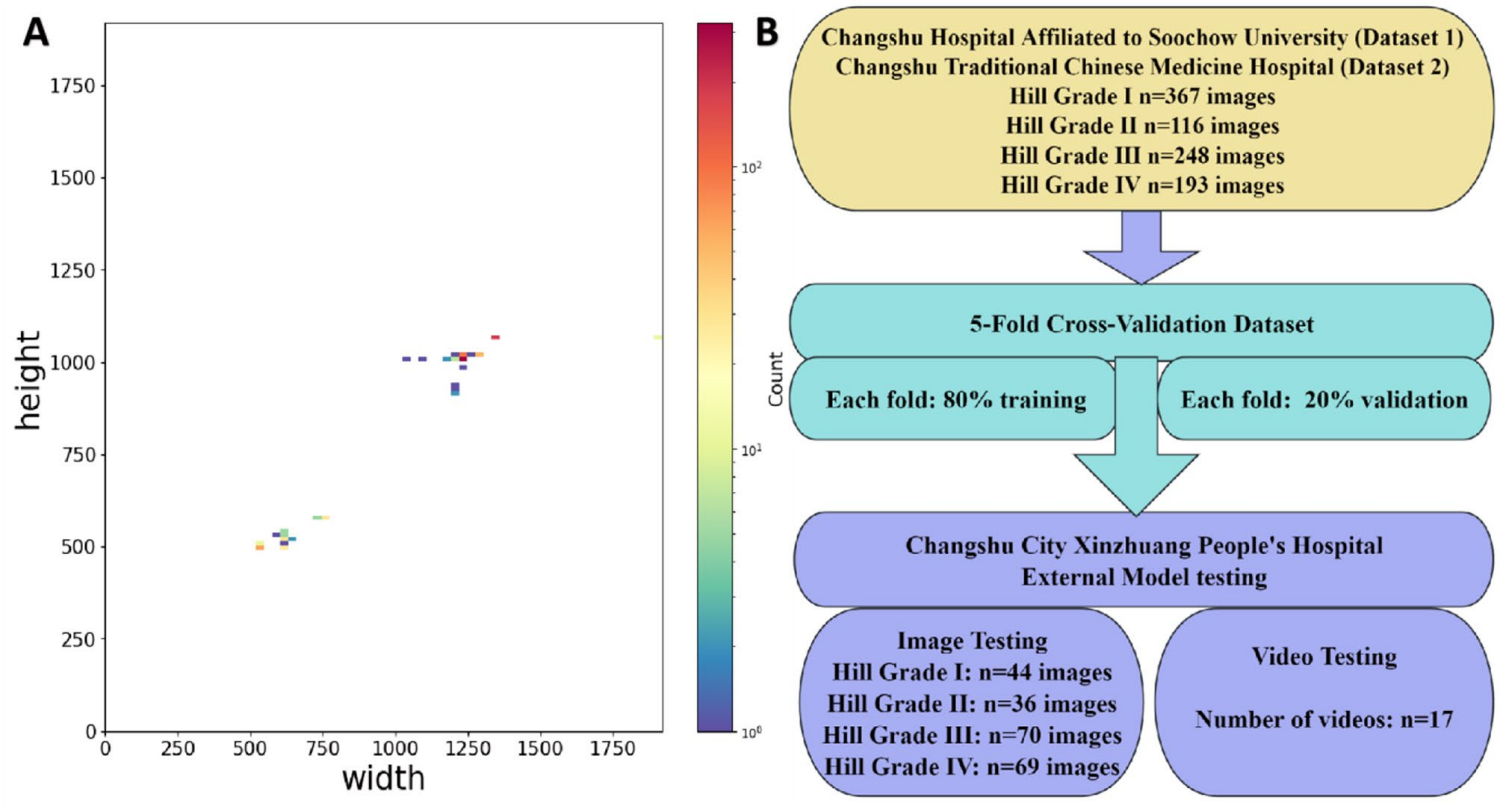
**A**: Distribution of image sizes in the dataset. **B**: Distribution of various categories of images across different datasets

In this study, we utilized four datasets. Dataset 1 and Dataset 2 were collected from Changshu Hospital Affiliated with Soochow University and Changshu Traditional Chinese Medicine Hospital, comprising a total of 924 esophagogastric flap valve (GEFV) images. These datasets were used for model training with a five-fold cross-validation strategy. Dataset 3, obtained from Xinzhuang People’s Hospital of Changshu, contained 219 GEFV images and served as an independent external static image test set, without participating in the cross-validation process. Dataset 4, also sourced from Xinzhuang People’s Hospital of Changshu, consisted of 17 endoscopic videos and was designated for external video testing to ensure an independent evaluation of the model. To maintain the independence and validity of testing, the external test datasets were exclusively used for performance evaluation and were not involved in model training, cross-validation, or hyperparameter tuning. The endoscopic images and videos were acquired using Olympus endoscopes (GIF-HQ290, GIF-Q260J, GIF-H260Z, GIF-Q260; Olympus Medical Systems Corp., Tokyo, Japan) and Olympus endoscopic video systems (EVIS LUCERA ELITE CV-290/CLV-290SL and EVIS LUCERA CV-260SL/CLV-260SL). All collected images were anonymized prior to analysis. The dataset included GEFV images representing all four Hill classification grades (I to IV), covering a range of morphological variations. Representative images are shown in Fig. 1. Figure 2A illustrates the distribution of image dimensions within the dataset, where yellow indicates a higher concentration of images of the same size, while purple indicates lower concentrations. Figure 2B presents the dataset partitioning and class distribution.

### Image labelling

In this study, we adhered strictly to inclusion and exclusion criteria, collecting gastroesophageal flap valve (GEFV) endoscopic images from patients who underwent endoscopic examinations between January 2018 and October 2023. All images were required to clearly display the GEFV structure for Hill classification assessment. The exclusion criteria included patients with a history of esophageal, gastric, or thoracic surgery; patients with digestive system diseases such as gastrointestinal tumors, esophageal varices, peptic ulcers, or those with infectious esophagitis or eosinophilic esophagitis; patients with primary or secondary severe esophageal motility disorders (e.g., achalasia, scleroderma); as well as pregnant women, patients with severe cardiopulmonary diseases, coagulation disorders, or others unable to tolerate the examination. All endoscopic examinations were performed in the endoscopy center by professionally trained and certified endoscopists. These endoscopists also participated in systematic training and mock tests related to Hill classification. To ensure the comprehensiveness and quality of the examination, each gastroscopy lasted at least 7 minutes, ensuring at least 38 clear images of various parts of the upper digestive tract were obtained. These stringent standards and procedures aimed to enhance the quality and reliability of data collection, thereby ensuring the accuracy and validity of the study’s results.

**Fig. 3:**
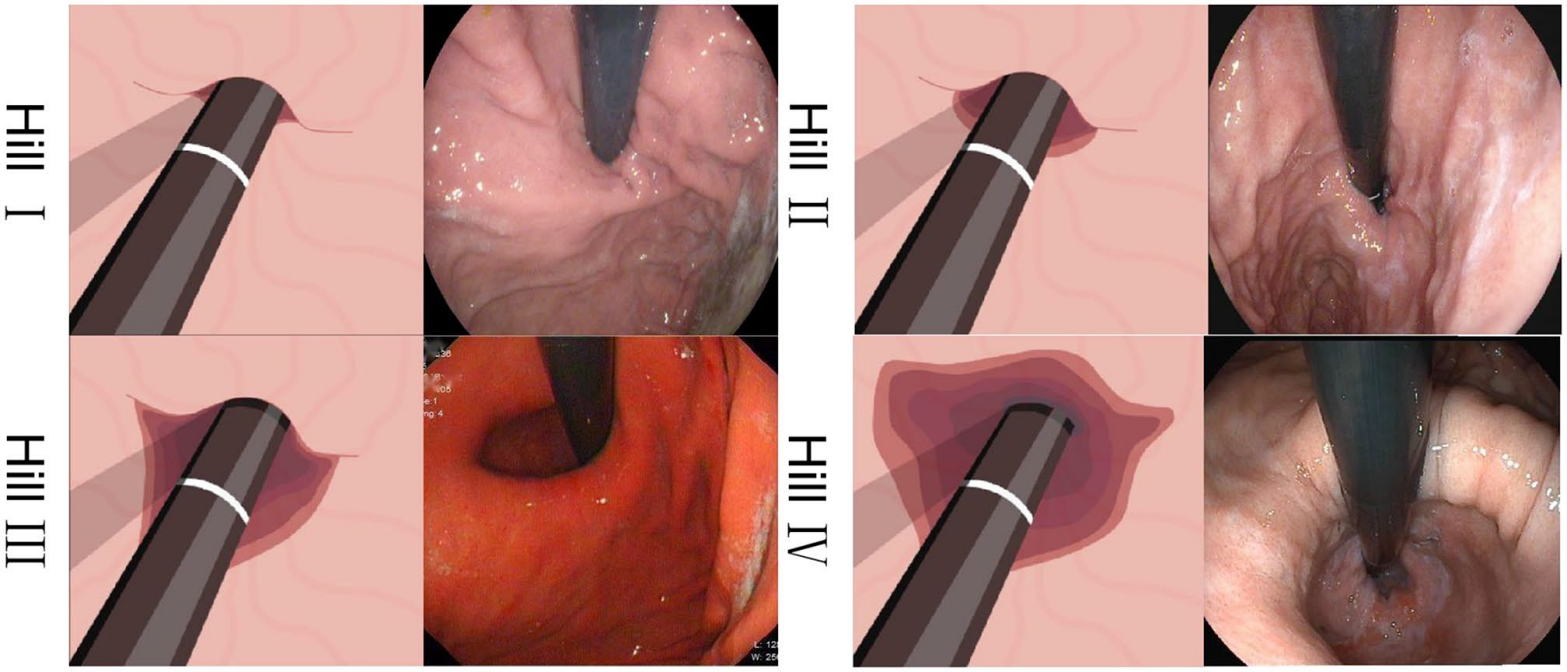
Reference image for the Hill classification criteria

Hill classification criteria [7]. Hill Grade I: The gastroesophageal flap valve is prominently defined, tightly wrapping around the endoscope along the lesser curvature. Hill Grade II: The valve is not as prominent as in Grade I and may occasionally not close completely due to respiration. Hill Grade III: The valve is almost non-existent, unable to tightly envelop the endoscope. Hill Grade IV: There is a complete absence of the valve. The gastroesophageal area is open, with the esophageal squamous epithelium easily visible, as shown in Fig. 3.

**Fig. 4:**
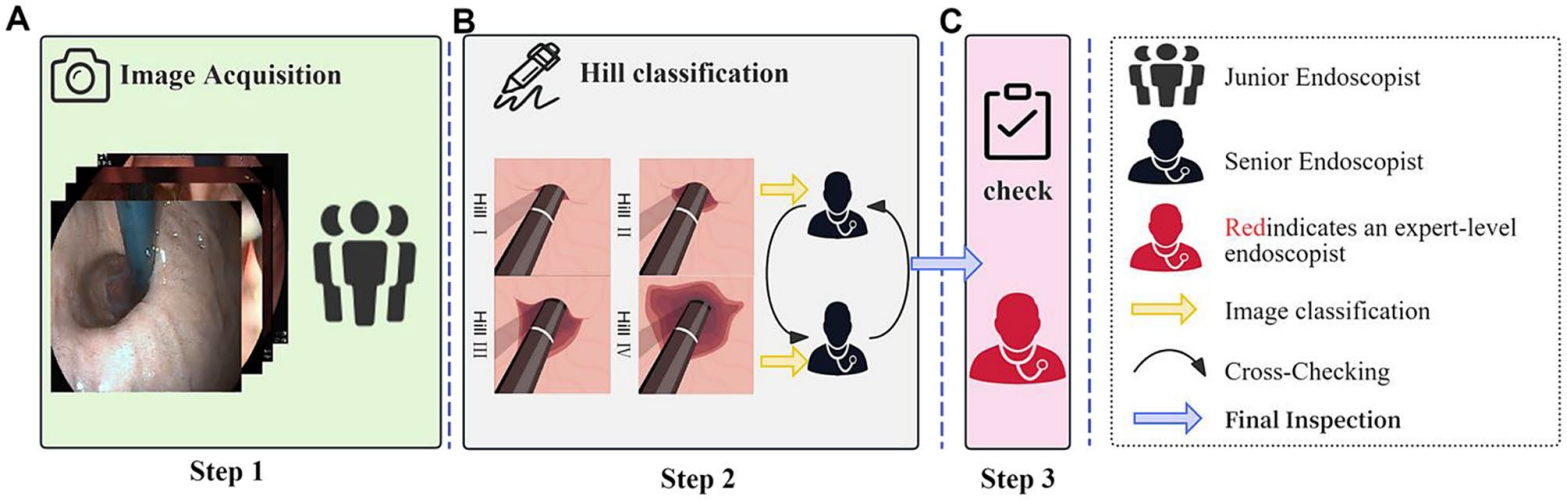
Image Labelling Process. (**A**) Step 1: A junior endoscopist captures gastroesophageal flap valve images in a retroflexed view from individuals of different ages and genders. (**B**) Step 2: Two senior endoscopists classify the images based on the Hill classification criteria and perform cross-checking. (**C**) Step 3: An expert-level endoscopist reviews the labels and makes the final decision

Three levels of endoscopists participated in this study. Expert endoscopists: with over 20 years of experience in the field of digestive endoscopy. Senior endoscopists: with 5–10 years of experience in digestive endoscopy. Junior endoscopists: with 1–3 years of experience in digestive endoscopy. To ensure the quality of the images and the accuracy of the grading, the labellers were divided into three teams, each responsible for a specific stage of the process. Only images that underwent labelling and verification following this workflow (Fig. 4) were included in the AI model training. All endoscopists involved in data annotation did not participate in the external testing process, thereby reducing the risk of bias.

### Deep learning network

#### Image preprocessing

To enhance the model’s generalization ability, this study systematically preprocessed and augmented the image data. Online data augmentation (real-time data augmentation) methods [16] were used, performing all data augmentation operations in real-time during the training process. This approach avoids generating new image files, ensuring the model is exposed to slightly different versions of images each time it trains. Specifically, for the training set, images were randomly resized and then cropped to 224 × 224 pixels, with random horizontal flipping added to increase data diversity. The image format was converted from PIL Image or numpy.ndarray to PyTorch Tensor and normalized to the [0, 1] range. Moreover, the RGB channels of the images were standardized, using means of [0.485, 0.456, 0.406] and standard deviations of [0.229, 0.224, 0.225]. The processing method for the test set was similar, but the short edge of the images was first adjusted to 256, then center-cropped to 224 × 224 pixels. The image format conversion, normalization, and RGB channel standardization for the test set were the same as for the training set. All preprocessing and augmentation operations were implemented using the torchvision library of PyTorch.

#### Model training configuration

To achieve image classification, transfer learning was utilized with pretrained models based on CNN and Transformer architectures. In the CNN segment, ResNet50 [17], VGG19 [18], DenseNet121 [19], and EfficientNet [20] models were selected; while in the Transformer segment, ViT [21], Swin Transformer [22], and CvT [23] models were chosen. These CNN models all include convolutional layers, average pooling layers, and fully connected layers with ReLU activation. To better adapt to our dataset, two dense layers with ReLU activation and an output layer with Softmax activation for classification were added to each pretrained model. The number of features in the output layer was set to four to fit our classification task.

All models were trained using the cross-entropy loss function, with different optimizers applied based on the model architecture. Specifically, CNN models primarily used Adam, VGG19 was trained with SGD, while Swin Transformer and CvT employed AdamW. During training, all models adopted the StepLR learning rate scheduling strategy, reducing the learning rate by 50% (gamma = 0.5) every 5 epochs. Additionally, an Early Stopping mechanism was implemented with a patience value of 8, meaning training was halted if the validation loss did not decrease for 8 consecutive epochs. The batch size was adjusted according to the computational requirements of each architecture. Detailed hyperparameter settings are provided in Table 1. For data augmentation, CNN models underwent random cropping and horizontal flipping, while Transformer models were further enhanced with up to 15° of random rotation. All operations were performed using the PyTorch framework.

**Table 1:** Model hyperparameter settings

Model	Learning Rate	Optimizer	Learning Rate Scheduler	Batch Size	Epochs	Patience
EfficientNet	1e^−4^	Adam	StepLR	60	80	8
ResNet50	1e^−4^	Adam	StepLR	60	80	8
VGG19	1e^−3^	SGD	StepLR	60	80	8
DenseNet121	1e^−3^	Adam	StepLR	60	80	8
ViT	1e^−3^	Adam	StepLR	32	80	8
Swin Transformer	1e^−5^	AdamW	StepLR	64	80	8
CvT	1e^−5^	AdamW	StepLR	64	80	8

#### Model performance evaluation

Deep learning models often exhibit a "black box" characteristic in medical image analysis, where the inputs and outputs are visible, but the internal decision-making mechanism is difficult to interpret. To address this, we employed advanced interpretability methods, including Gradient-weighted Class Activation Mapping (Grad-CAM) and SHAP (Shapley Additive exPlanations) techniques [24, 25], to enhance the transparency and interpretability of the model’s decision-making process. The Grad-CAM method utilizes feature maps from convolutional layers and gradient information to generate heatmaps, visually highlighting the areas in the image that contribute most to the model’s predictions. This approach allows us to visualize and understand the model’s focus areas when identifying different Hill grades, enabling a deeper analysis of the model’s attention mechanism. On the other hand, the SHAP technique calculates the contribution of each feature to the model’s output, providing a detailed explanation for each prediction. This method incorporates the concept of Shapley values from classical game theory, offering a powerful tool for understanding how models process complex medical image data.

In this study, we utilized deep learning to automate the Hill classification of the gastroesophageal flap valve, including Hill Grades I, II, III, and IV. To explore the model’s semantic classification capability, we extracted the intermediate layer outputs of the image classification model as semantic features. By registering forward hooks to the target layer, we captured these features. Subsequently, we employed the t-SNE method to reduce these high-dimensional features to a two-dimensional space [26]. For further analysis of these reduced features, we used the Plotly library for interactive visualization. First, a DataFrame containing the t-SNE reduction results was created, which included two-dimensional coordinates, the original data’s labeled categories, predicted categories, and image paths. Then, we utilized Plotly’s scatter function to create a scatter plot that displays each category with different colors, labels, and symbols, allowing users to view the detailed image paths of each point by hovering over them. Additionally, we optimized the chart’s appearance and saved this interactive graphic as an HTML file for detailed exploration and analysis in a web browser.

To ensure the optimal selection of models for external validation, we predefined performance thresholds in our study design to guarantee both high classification accuracy and computational efficiency for real-time clinical application. The specific criteria included an internal test set accuracy of ≥80%, an F1-score of ≥0.80 to ensure balanced classification performance for Hill grading, and a computational efficiency of ≥50 FPS to meet real-time inference and clinical usability requirements.

#### Multi-device terminal model deployment

**Fig. 5:**
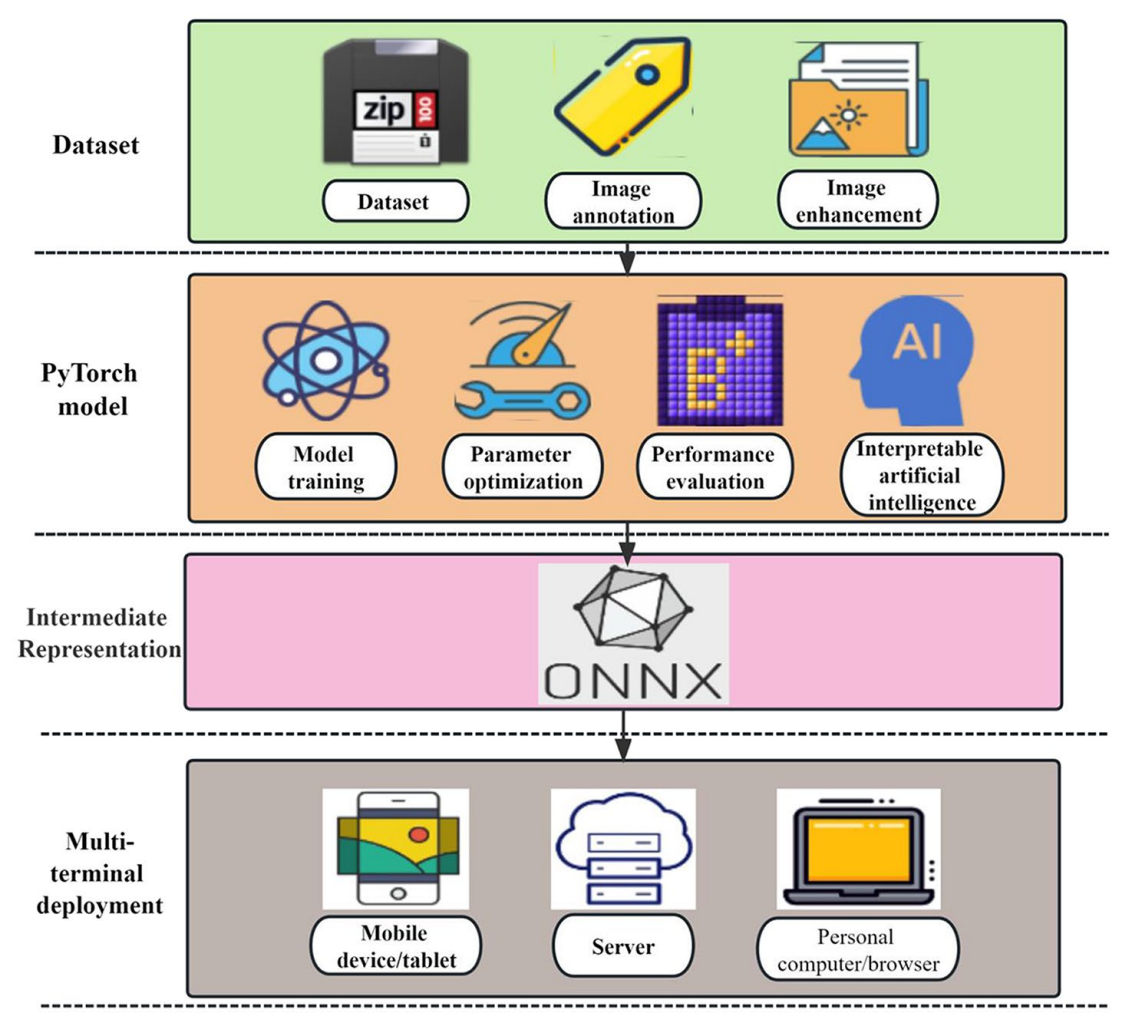
The complete workflow of model development and deployment; ONNX is an open file format designed to facilitate model interoperability across various deep learning frameworks, thereby simplifying the deployment and operation of models on diverse platforms and hardware

To automate the implementation of Hill classification of gastroesophageal flap valve (GEFV) morphology, we developed a deep learning model and deployed it on various devices, including desktop computers, laptops, and online browsers at an endoscopy center. This model is designed to provide real-time and accurate monitoring for gastroscopy videos, fulfilling the minimum frames per second (fps) requirements for real-time inference prediction. During its development, transfer learning techniques were utilized, and the model, based on PyTorch, underwent specific optimizations. For cross-platform deployment, the model was converted to the Open Neural Network Exchange format (ONNX). Utilizing ONNX Runtime, the model efficiently operates on different operating systems like Linux, Windows, and MacOS, and is optimized for various hardware (CPU, GPU). ONNX, as an open standard for deep learning [27], not only ensures model interoperability but also expands deployment options, thereby enhancing the real-time recognition accuracy of GEFV morphology in gastroscopy videos. The entire process of model development and deployment is detailed in Fig. 5.

### Experimental platform and statistical analysis

In this study, we employed a computer equipped with an RTX 3080 graphics card (10.5GB VRAM), a 6-core E5-2680 v4 CPU, and 500GB of disk space. Utilizing Python libraries such as PyTorch (1.10.0 + cu113) and OpenCV (4.5.4.60), we efficiently built and trained deep learning models and processed images. Data organization, analysis, and visualization were conducted using Pandas (1.3.4), NumPy (1.21.4), Matplotlib (3.5.0), and Plotly (5.4.0). Model saving and loading were facilitated by H5py (3.6.0).

To thoroughly evaluate the model’s performance in image classification tasks, a diverse array of evaluation metrics was adopted. Firstly, accuracy (Acc), calculated as Accuracy = (TP + TN)/(TP + FP + FN + TN), reflects the model’s overall accuracy in all predictions. Secondly, precision, calculated as Precision = TP/(TP + FP), measures the accuracy of the model in predicting positive classes. Recall, calculated as Recall = TP/(TP + FN), indicates the model’s ability to identify positive classes. The F1 score, calculated as F1 = 2 * (Precision * Recall)/(Precision + Recall), considers both precision and recall. Specificity, calculated as Specificity = TN/(TN + FP), measures the model’s ability to identify negative classes. Average precision (AP) assesses the mean accuracy at different thresholds. The area under the curve (AUC) reflects the model’s ability to distinguish between positive and negative classes. The Matthews correlation coefficient (MCC), calculated as MCC = [(TP * TN) − (FP * FN)]/sqrt[(TP + FP) * (TP + FN) * (TN + FP) * (TN + FN)], is a comprehensive performance metric. A confusion matrix was used for quantitative analysis of the model’s classification predictions. Lastly, frames per second (fps), calculated as fps = 1/(average processing time), measures the model’s inference prediction speed. Furthermore, Cohen’s κ (Kappa) statistics were employed to assess the inter-rater agreement between endoscopists in their diagnostic evaluations. Cohen’s κ accounts for the agreement occurring by chance, and its values range from −1 (complete disagreement) to 1 (perfect agreement), with higher values indicating stronger consistency. In this study, predictions with a confidence level of 80% or higher were considered highly reliable, representing the model’s dependable predictions. McNemar’s test was used to compare the diagnostic accuracy between the AI model and endoscopists, with a *p*-value < 0.05 considered statistically significant.

## Results

### Baseline data

During the study period, a total of 1160 patients’ data, including 1143 GEFV images and 17 gastroscopy videos, were included in the research. Among these patients, 56.7% (658 individuals) were male, and 42.3% (502 individuals) were female, with an average age of 63.2 ± 26.5 years. Regarding gastroesophageal reflux disease (GERD), 28.5% of the patients (331 individuals) did not exhibit GERD symptoms, 33.0% were diagnosed with non-erosive reflux disease (383 individuals), and 38.4% suffered from reflux esophagitis (446 individuals). GERD, Non-erosive reflux disease (NERD), and Reflux esophagitis (RE) were diagnosed based on standardized criteria. GERD followed the Lyon Consensus, incorporating symptoms, esophageal pH monitoring, and response to proton pump inhibitors. RE was classified using the Los Angeles system, while NERD was defined as GERD symptoms without mucosal damage on endoscopy. Diagnoses were extracted from electronic medical records and confirmed by at least two board-certified gastroenterologists, with discrepancies resolved by consensus. Further details on the patients’ baseline data are provided in Table 2.

**Table 2:** Baseline data of the patients

Variable	Overall(n = 1160)
Sex	
man	658(56.7%)
female	502(42.3%)
Age(years)	63.2 ± 26.5
GERD	
No	331(28.5%)
NERD	383(33.0%)
RE	446(38.4%)
Hill classification	
Grade I	411 (35.8%)
Grade II	152 (13.4%)
Grade III	318 (27.8%)
Grade IV	262(22.9%)

### Performance comparison of various deep learning models

**Fig. 6:**
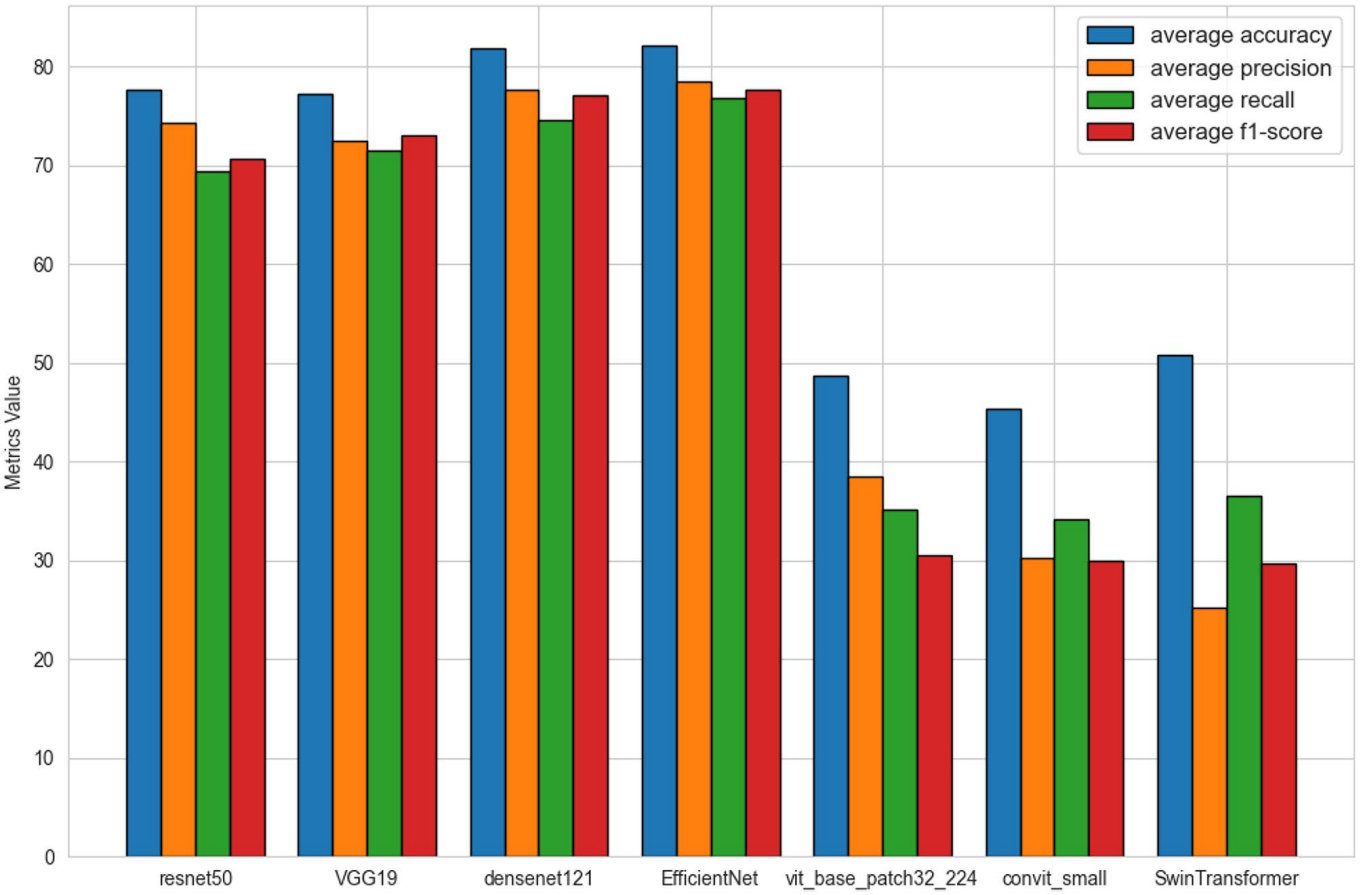
Comparison of the average performance of different deep learning (DL) models on the test dataset

In this study, a total of 924 colonoscopy images were utilized, with 738 allocated to the training set and 186 to the test set. We conducted fine-tuning through transfer learning based on pretrained models from two major deep learning architectures: CNN and Transformer. Within the CNN architecture, models such as EfficientNet, ResNet50, Densenet121, and VGG19 were adopted, while in the Transformer architecture, models like vit_base_patch32_224, SwinTransformer, and convit_small were selected. To accommodate the four-class labeling of GEFV morphology Hill grading, adjustments were made to the output layers of these models. During training, the Adam optimizer was used for efficiency. A comparison of these models’ performance metrics on the test set is illustrated in Fig. 6.

In the automatic Hill classification of GEFV morphology, EfficientNet demonstrated the best performance with a test average accuracy of 82.03%, surpassing ResNet50 (77.65%) and Densenet121 (81.77%). The average precision, recall, and F1 score of EfficientNet reached 78.43%, 76.84%, and 77.56%, respectively, highlighting its robustness and high accuracy in this task. In comparison, although Transformer models overall did not perform as well as CNNs, SwinTransformer among them had the highest average accuracy in the Transformer category, achieving 50.86%, proving its potential value in this field.

### Model training and evaluation results

**Fig. 7:**
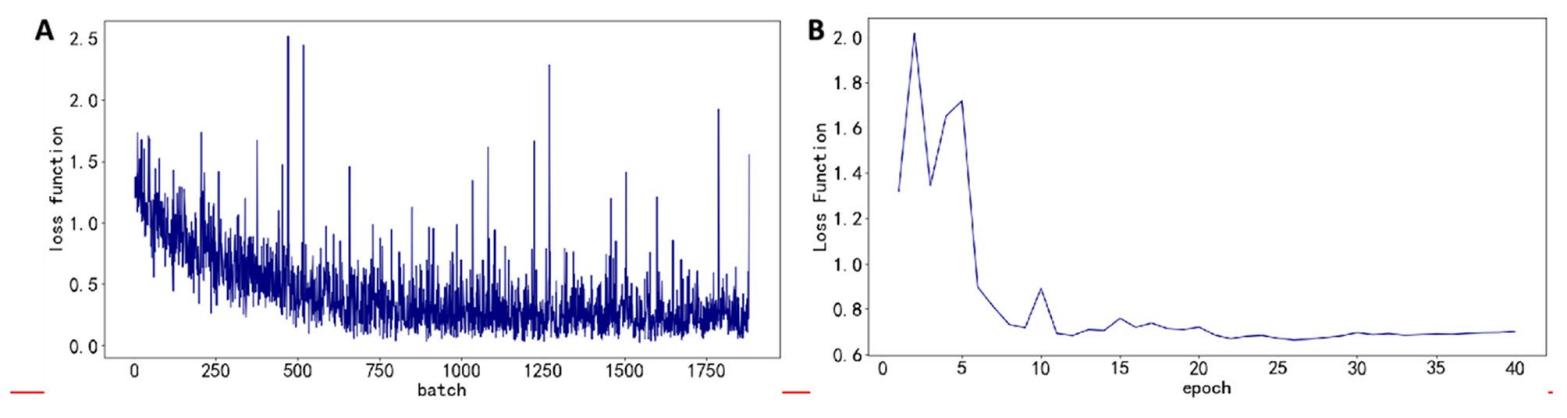
Trends in the average loss function of the EfficientNet-Hill model during training and testing. **A**: Change in the average loss function on the training set; **B**: Change in the average loss function on the test set

**Fig. 8:**
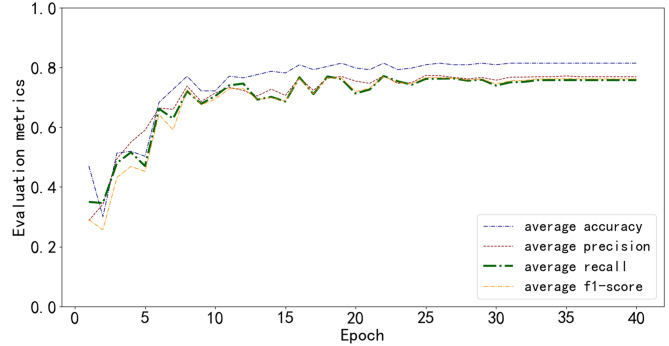
Trends in various classification evaluation average metrics of the EfficientNet-Hill model during training. The horizontal axis represents the training epochs

Figure 7A illustrates the evolution of the loss function during training of the high-performing deep learning model EfficientNet (hereafter referred to as EfficientNet-Hill) using five-fold cross-validation. The training data were partitioned into five subsets, with one subset serving as the validation set and the remaining four used for training in each fold; the resulting metrics were then averaged and plotted. It is evident that the loss continuously declines with increasing iterations and eventually stabilizes, indicating steady model convergence. After 35 epochs, the average loss on the validation set reached 0.66, as shown in Fig. 7B, while the average accuracy attained 82.03%, underscoring its exceptional classification performance. Figure 8 further depicts the average trends of accuracy, precision, recall, and F1 score across the folds during training, providing a clear view of the model’s overall stability and performance trajectory.

### Model predictive performance on external test set

Utilizing a dataset from the Changshu Hospital Affiliated to Soochow University and the Changshu Traditional Chinese Medicine Hospital (*n* = 924), we developed the EfficientNet-Hill deep learning model aimed at automating Hill classification for various gastroesophageal flap valve (GEFV) morphologies. To verify the model’s generalization capabilities, 219 GEFV images from the Changshu Xinzhuang People’s Hospital were used as an independent external test set. This independent validation aids in thoroughly assessing the model’s predictive performance in real-world scenarios, while also preventing overfitting issues.

**Fig. 9:**
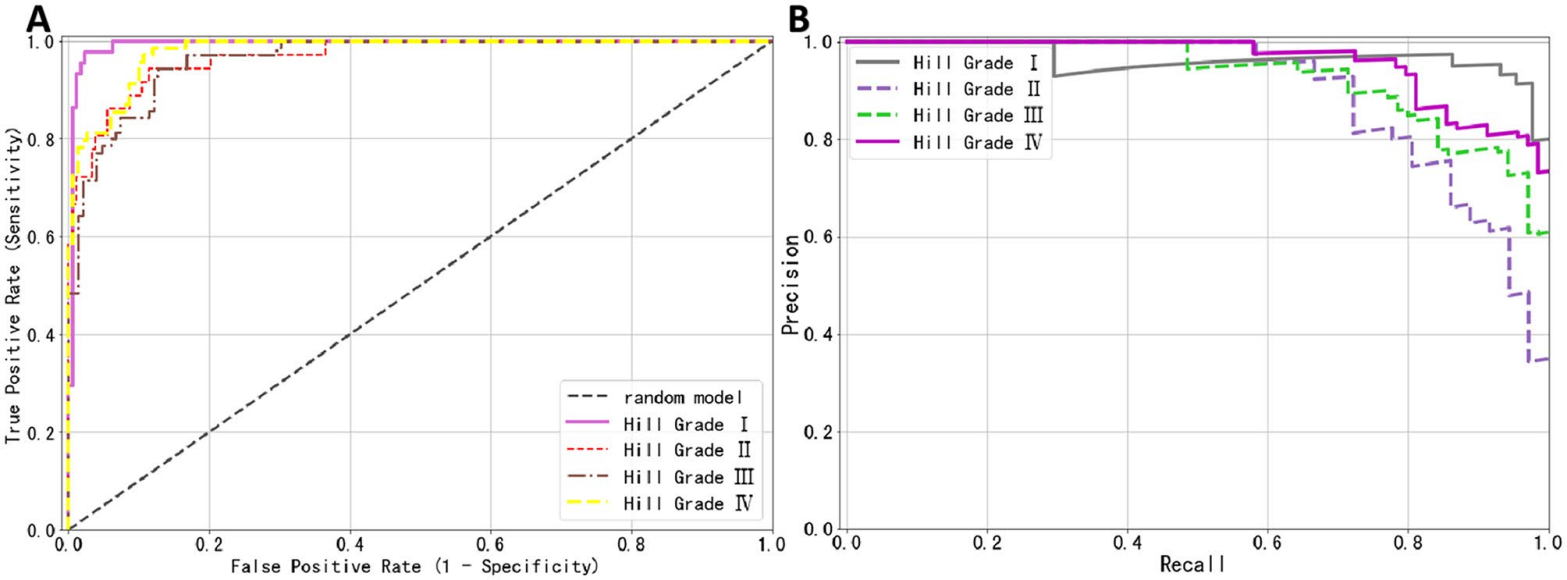
Predictive performance of the model on the external test set. (**A**) Receiver operating characteristic (ROC) curve; (**B**) Precision-recall (PR) curve

The EfficientNet-Hill deep learning model demonstrated exceptional automated classification performance on the external test set. Particularly notable were its results in Hill Grade I and Hill Grade IV categories, where it achieved AUC values of 0.994 and 0.980 respectively, showcasing its high discriminative capability. For Hill Grade II and Hill Grade III categories, the AUCs were 0.969 and 0.964, as shown in Fig. 9A. Overall, the model’s macro average precision was 0.848, with a sensitivity of 0.833 and an AUC of 0.977. The weighted averages for precision, sensitivity, and AUC were 0.840, 0.836, and 0.976 respectively, as detailed in Table 3.

**Table 3:** Classification performance of EfficientNet-Hill on the external test set

Category	precision	recall(sensitivity)	specificity	f1-score	accuracy	AP	AUC	MCC
Hill I;	0.951	0.886	0.989	0.918	0.886	0.966	0.994	0.899
Hill II	0.824	0.778	0.967	0.800	0.778	0.901	0.969	0.762
Hill III	0.769	0.857	0.879	0.811	0.857	0.931	0.964	0.717
Hill IV	0.848	0.812	0.933	0.83	0.812	0.959	0.980	0.754
macro avg	0.848	0.833	0.942	0.84	0.833	0.939	0.977	0.783
weighted avg	0.840	0.836	0.933	0.836	0.836	0.942	0.976	0.773

**Fig. 10:**
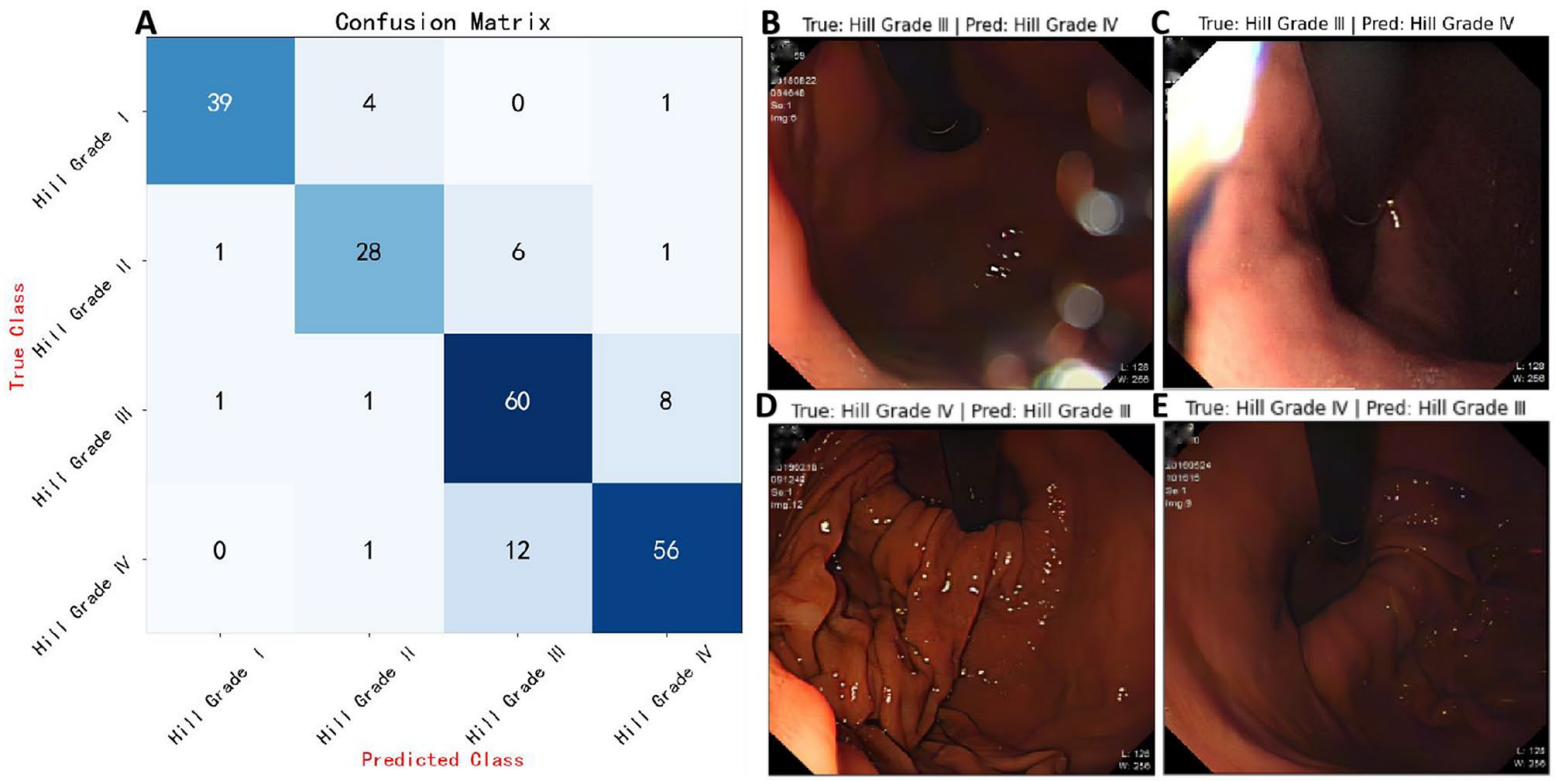
Performance of the model on the external test set. (**A**) Confusion matrix: demonstrating the model’s classification accuracy. (**B**) & (**C**) Image examples: two cases where the model incorrectly classified images with true labels of Hill Grade III as Hill Grade IV. (**D**) & (**E**) Image examples: two cases where the model incorrectly classified images with true labels of Hill Grade IV as Hill Grade III

As illustrated in the precision-recall (PR) curve in Fig. 9B, the model developed in this study exhibited excellent performance across different Hill grading categories. For the Hill Grade I category, the model showed high precision (0.951) and a good recall rate (0.886), approaching an ideal state. In the Hill Grade II category, although precision slightly decreased to 0.824, the recall rate remained at a high level (0.778), demonstrating the model’s effective recognition ability in this category. For Hill Grade III, the model maintained its stability, with precision and recall rates of 0.769 and 0.857, respectively. In the Hill Grade IV category, while the recall rate slightly dropped to 0.812, precision increased to 0.848, maintaining high accuracy. Overall, the model’s macro average and weighted average precision were 0.848 and 0.840, respectively, with recall rates of 0.833 and 0.836, and the average precision (AP) for each category exceeded 0.900, further evidencing the model’s robustness and consistency across different categories. Additionally, through the analysis of the confusion matrix (as shown in Fig. 10A), we further confirmed the model’s classification accuracy and robustness in each Hill grading category.

In this study, although the deep learning (DL) model exhibited impressive performance in most cases, there were also some notable classification errors. Specifically, as illustrated in Fig. 10 and (C), some images marked as Hill Grade III were incorrectly predicted as Hill Grade IV. Conversely, as shown in the two cases in Fig. 10 and (E), the model misclassified images that were actually Hill Grade IV as Hill Grade III. These classification discrepancies may stem from overlapping features between image categories, unexpected reflections, excessive shooting distances, and image blurriness, among other factors.

**Fig. 11:**
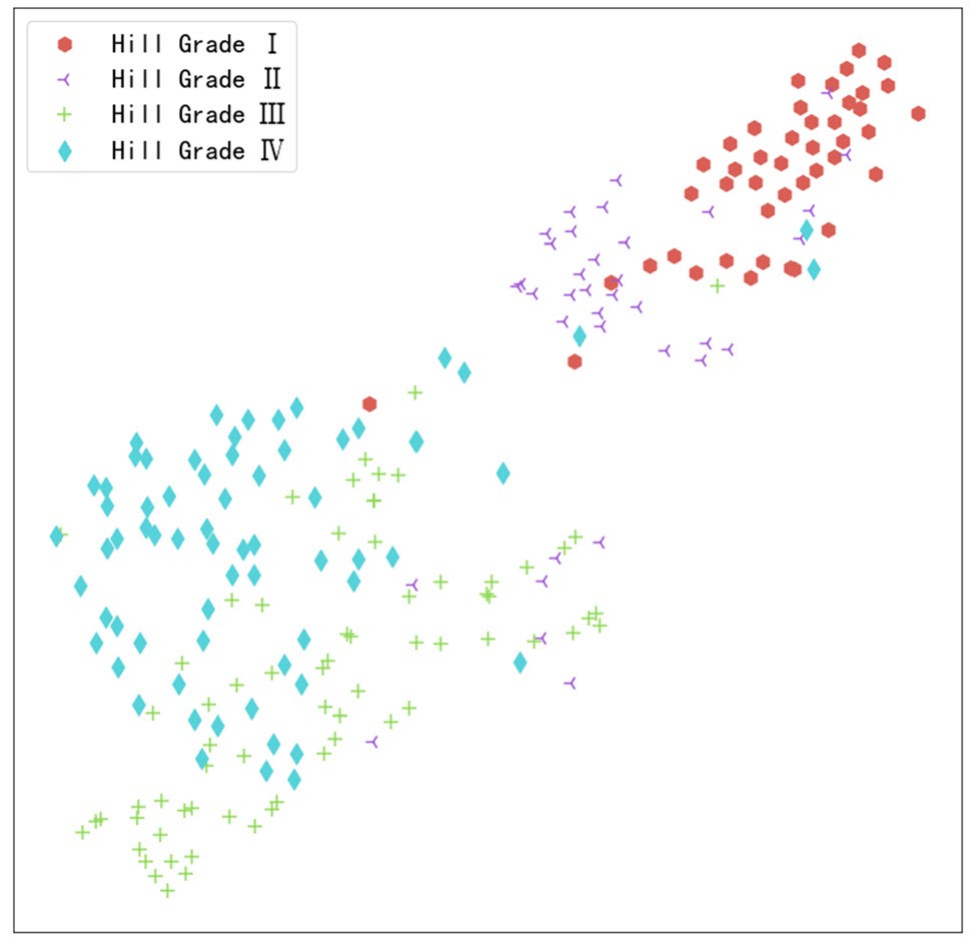
Two-dimensional semantic feature map of images from the external test set

In our study, to uncover underlying patterns and optimize analysis, we employed t-SNE technology to reduce high-dimensional image features to two dimensions. The results displayed in Fig. 11 show some overlap between green plus signs (representing Hill Grade III) and blue diamonds (representing Hill Grade IV). This visual overlap explains why the model experiences misclassification between these two categories. It suggests that although these two categories are distinguishable in the high-dimensional feature space, their features still bear certain similarities in the reduced two-dimensional space, leading to classification challenges for the model. Additionally, the orange circles in the graph (representing Hill Grade I) are semantically distant from the green plus signs (representing Hill Grade III), indicating that the model performs well in differentiating these two categories.

We further utilized Plotly Express to create an interactive scatter plot showcasing the data reduced by t-SNE technology. This scatter plot has been saved as an HTML file, which can be visualized and analyzed through a browser. The file has been uploaded online and can be downloaded via the provided link (https://share.weiyun.com/5fIIegtm). Once users download this HTML file, they can intuitively explore the data and model features through clicking operations. This interactive visualization tool enables users to gain a deeper understanding of the data structure and model performance, facilitating a comprehensive comprehension of the model’s classification capabilities.

### Deep learning vs. endoscopists diagnostic performance

**Fig. 12:**
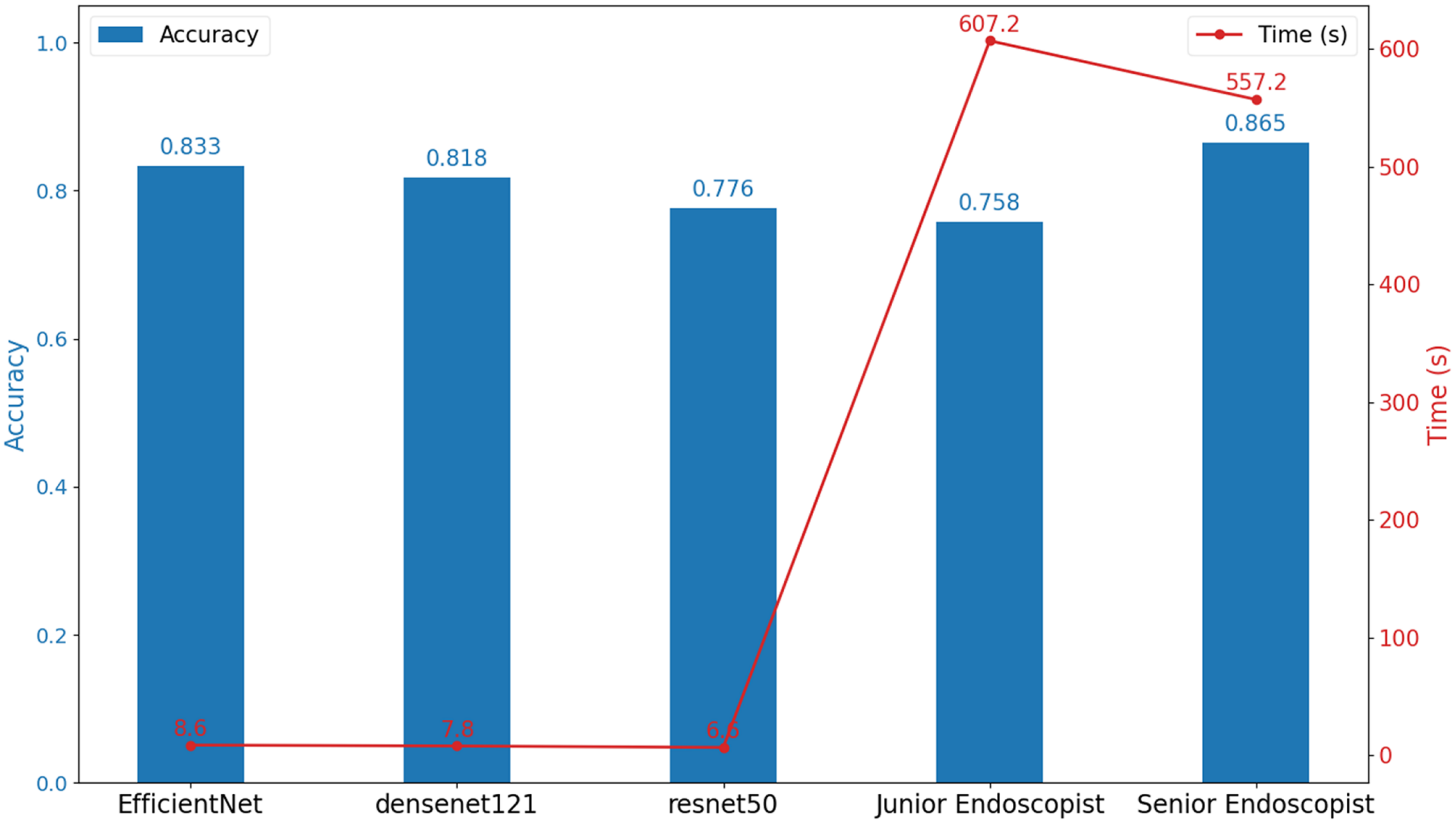
Comparison of hill classification accuracy and time between different deep learning models and endoscopists of varying experience. The bar graph represents accuracy comparison, while the line graph shows time comparison (in seconds)

In this study, we selected the top three performing deep learning models for performance evaluation on an external test set containing 219 gastroesophageal flap valve (GEFV) images. For comparative analysis, two senior endoscopists and two junior endoscopists independently assessed these images, as illustrated in Fig. 12. The inter-rater agreement among the endoscopists was evaluated using Cohen’s κ (Kappa) statistics, yielding a value of 0.7805 for senior endoscopists and 0.7132 for junior endoscopists.The EfficientNet and Densenet121 models demonstrated significant accuracy in the automated Hill grading task, with accuracy rates of 0.833 and 0.818 respectively. This was notably superior to the accuracy of junior endoscopists (0.758), but slightly lower than that of senior endoscopists (0.865). In addition, McNemar’s test was conducted to statistically compare the classification performance between the deep learning models and the endoscopists. The test revealed a statistically significant difference between the models and the junior endoscopists (*χ²* = 8.50, *p* = 0.0036), whereas no significant difference was observed between the models and the senior endoscopists (*χ²* = 0.26, *p* = 0.6069). In terms of the time required for image recognition, these models also showed significant efficiency advantages, requiring only 6.6 to 8.6 seconds, compared to the much longer times required by endoscopists (senior 557.2 seconds, junior 607.2 seconds). The deep learning models significantly outperformed doctors in image recognition speed, providing strong evidence for the use of artificial intelligence in assisting medical image analysis.

**Fig. 13:**
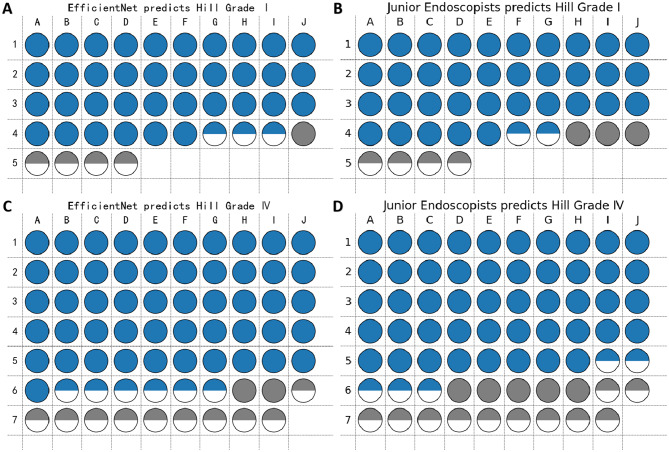
Comparison of predictive results between the EfficientNet model and junior endoscopists on the external test set at different confidence levels. Each circle represents a GEFV image, where green and gray signify correct and incorrect predictions, respectively. Solid circles indicate high-confidence predictions, while half circles represent low-confidence predictions

In this study, we recorded each endoscopist’s judgment results and confidence levels and calculated the averages for comparison with the model’s performance. A confidence level of 80% or higher was considered high confidence, while values below this threshold were deemed low confidence, to ensure the reliability of predictions. Taking the EfficientNet-Hill model’s predictions for Hill Grade I category as an example, the model demonstrated the following performance: of the 36 Hill Grade I images, the model correctly predicted 36 with high confidence; only 1 Hill Grade I image was incorrectly predicted with high confidence. Additionally, there were 4 images incorrectly predicted with low confidence, and 3 images correctly predicted with low confidence. As illustrated in Fig. 13, using Hill Grades I and IV as examples, analyzing this visual representation allows for a better understanding of the differences in predictive accuracy and confidence between the model and endoscopists.

### Model interpretation

**Fig. 14:**
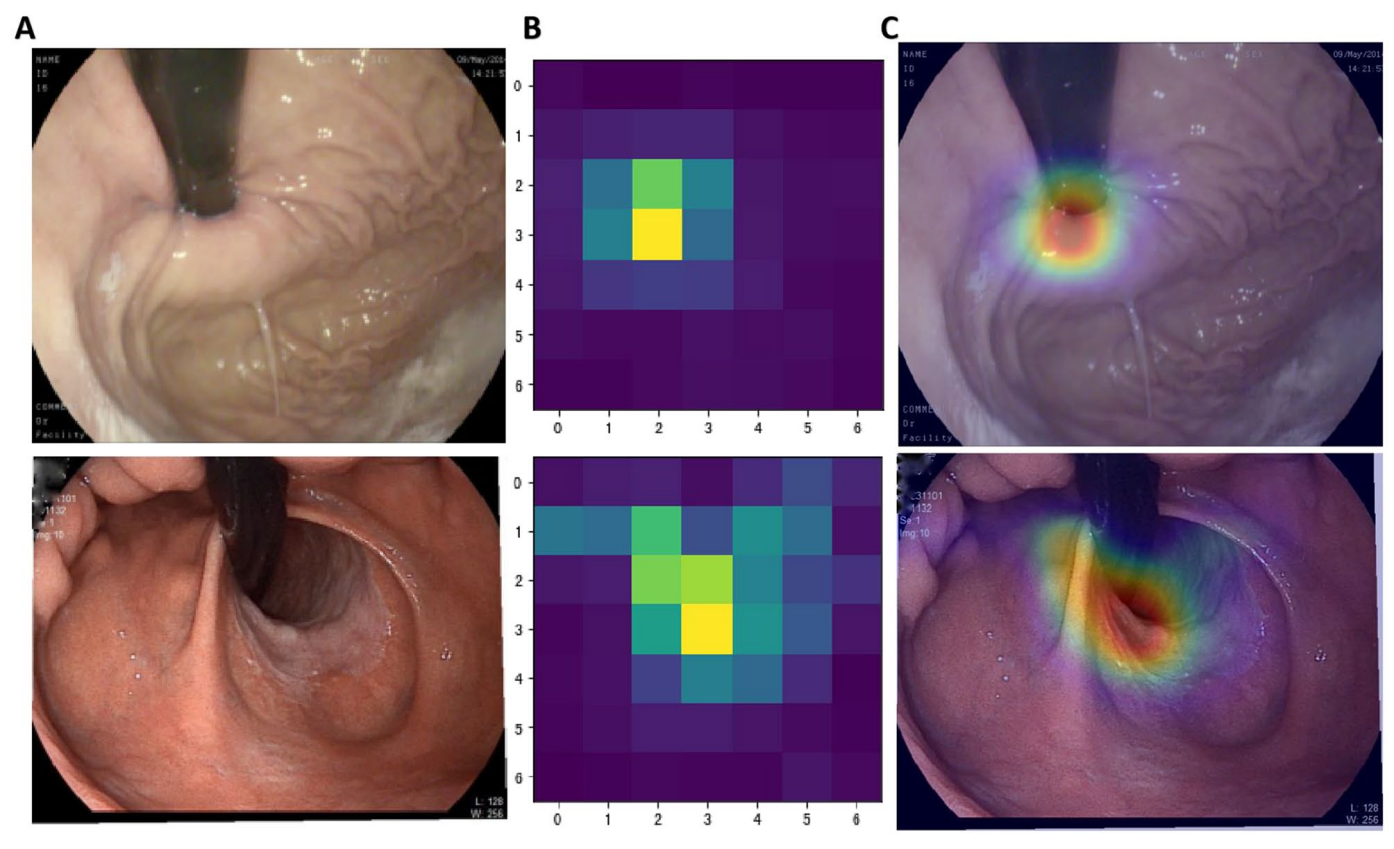
Interpretability analysis of the automated Hill grading model. Column **A** displays the original endoscopic images; Column **B** presents the pixel activation heatmaps generated using the Grad-CAM technique; and Column **C** illustrates the overlay of the original images with the activation heatmaps. The first row in the figure features images of Hill Grade I, while the second row displays original images of Hill Grade IV

To elucidate the decision-making mechanism of deep learning models in Hill classification, the torchcam library was utilized in conjunction with the Grad-CAM (Gradient-weighted Class Activation Mapping) visualization method. Figure 14 displays: Column A contains the original endoscopic images; Column B shows the pixel activation heatmaps based on feature extraction by EfficientNet-Hill, highlighting the key areas relied upon in the model’s decision-making; Column C presents the overlay of the activation heatmap on the original images, where warm areas (red regions) indicate the key pathological parts identified by the model, signifying higher weight assigned by the model during image classification judgment; lighter areas (such as yellow and blue) represent lower weight assigned by the model in the classification process.

**Fig. 15:**
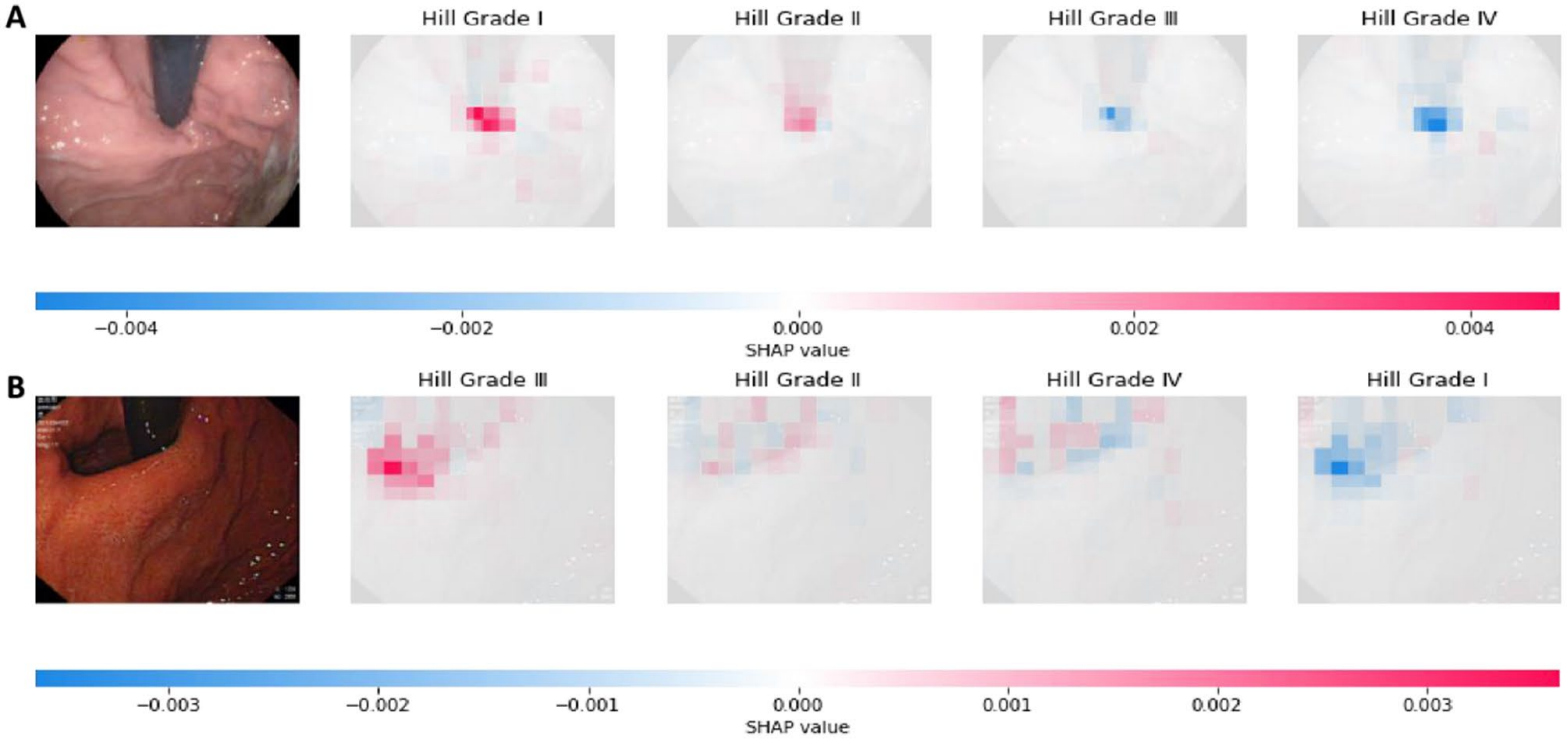
Interpretability analysis using SHAP. (**A**) SHAP visualization for the prediction of Hill Grade I; (**B**) SHAP visualization for the prediction of Hill Grade III. Red indicates a positive contribution to the prediction, while blue signifies a negative contribution. When the red areas are significantly more prominent than the blue areas, the image is predicted by the model to be of that category

To gain a deeper understanding of the model’s predictive logic, SHAP (SHapley Additive exPlanations) technology was employed for analysis. As shown in Fig. 15, Subfigure A and Subfigure B are real classifications of Hill Grade I and Hill Grade III, respectively. The intensity of the pixel colors in the figure reflects their contribution to the model’s prediction: red indicates a positive contribution, while blue signifies a negative contribution. In Subfigure A, the red areas are more prominent compared to the Hill Grade II, III, and IV categories, leading the model to accurately classify it as Hill Grade I. Similarly, Subfigure B is accurately predicted as Hill Grade III.

### Model-based video prediction and multi-terminal deployment

**Fig. 16:**
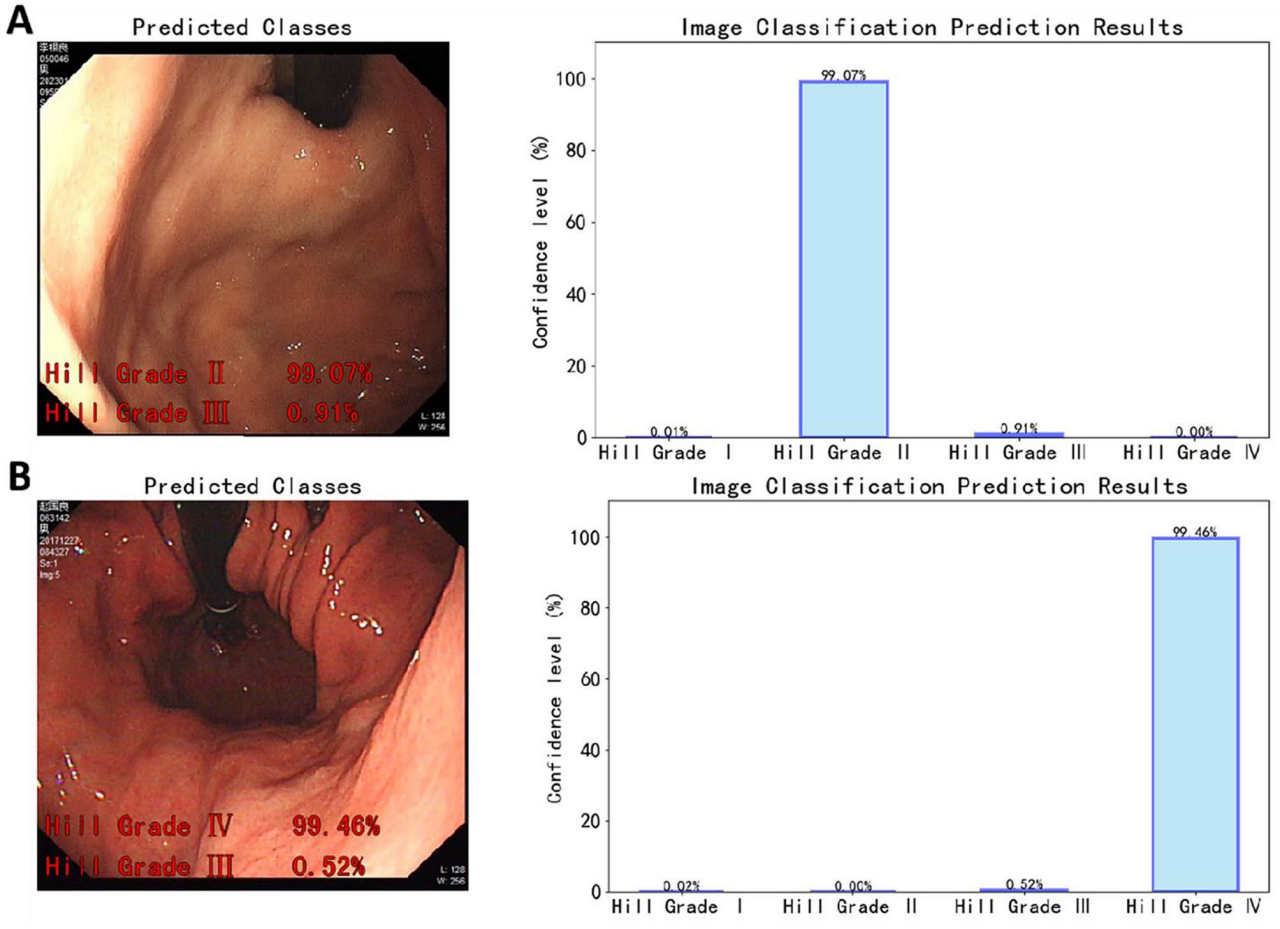
Predictive results and confidence levels after model deployment, with the left column showing the original images and the right column displaying corresponding predictive confidence bar charts. (**A**) A single frame from a Hill Grade II video. (**B**) A single frame from a Hill Grade IV video

To facilitate convenient deployment of the model on the inference engine, we employed transfer learning to train a PyTorch deep learning model and converted it into the ONNX (Open Neural Network Exchange) format. ONNX provides a standardized representation of the model, enabling AI researchers to easily deploy and share their findings across different platforms and devices. This conversion allows our EfficientNet-Hill model to be deployed in various environments (such as local computers, web frontends) for real-time automated Hill grading of gastroesophageal flap valves (GEFV). Utilizing the OpenCV library, each frame is captured in real-time from the video source and processed frame by frame through the ONNX model for inference. Figure 16 demonstrates the predictive results for a single frame image. On the left side of the image, the original scene displays the model’s predictions for the top two classifications and their corresponding confidence levels in red font at the lower left corner. The image on the right shows the confidence level bar charts for each classification. Subfigure A and Subfigure B respectively present the model’s predictions and confidence levels for single-frame images with true labels of Hill Grade II and Hill Grade IV.

To assess the performance of the EfficientNet-Hill model in real-world application scenarios, we randomly selected three videos from the external test set (Dataset 4) for demonstration. Table 4 provides links to these videos along with corresponding QR codes, making it convenient for users to access the videos via the links or watch them by scanning the QR codes. These videos intuitively demonstrate the process of the AI model automatically performing Hill grading on gastroesophageal flap valve (GEFV) videos on local terminal devices. Video 1 shows the model’s prediction process with real-time heatmap effects. This heatmap visualizes the key areas the model focuses on during prediction. Video 2 displays the real-time prediction of multiple GEFV images using a camera after the model is deployed on a local computer. Particularly noteworthy is the model’s high speed during inference prediction, averaging over 50 frames per second (fps).

**Table 4:** Real-time prediction of videos by the efficientnet-hill model

	Video Links
Online Streaming Link	Video 1: https://share.weiyun.com/mGY6cCHW Video 2: https://share.weiyun.com/IDswqAk4
Scan QR Code to Watch Video Online	

This video series (including Video 1 and Video 2) demonstrates the model's effectiveness in real-time Hill classification of gastroesophageal flap valves. In Video 1, the top left corner displays the model's predictions for the top two categories and their corresponding confidence levels in red font, while a real-time dynamic heatmap reveals the key areas of focus for the model. Video 2 showcases the process of real-time inferential prediction on multiple GEFV images displayed on an iPad, using a camera after the model is deployed on a local computer

## Discussion

In this study, we developed seven computer vision (CV) models based on deep learning (DL) to automate the Hill classification of gastroesophageal flap valve (GEFV) morphologies. Among these models, four are based on Convolutional Neural Network (CNN) architectures, while three utilize Transformer architectures. Utilizing gastroscope datasets provided by three large comprehensive hospitals in Jiangsu Province, China, we selected 1143 images and 17 videos, covering GEFV images with Hill Grades I, II, III, and IV features, for model development and testing. After validation through an external test set, the EfficientNet model emerged as the most outstanding. This model has been successfully deployed on multiple terminal devices, achieving real-time video prediction capabilities with an average inference speed of over 50 frames per second (fps). Comparisons with endoscopists of varying experience levels in diagnostic performance highlighted the potential of this model in clinical applications. Our research conducted a comparative assessment of CNN and Transformer in automated Hill grading, identifying the optimal model and covering the complete process from model development and testing to interpretability analysis and multi-end deployment.

In endoscopic diagnosis of gastroesophageal reflux disease (GERD) patients, it’s crucial to comprehensively consider different subtypes of GERD, potential complications, and other related anatomical abnormalities. This includes assessing the condition of the gastroesophageal flap valve (GEFV) and the presence of hiatal hernia. In the diagnostic process of GERD, patients presenting with reflux and heartburn symptoms may initially be diagnosed with GERD, but in reality, more than one-third of such patients do not exhibit the pathological characteristics of GERD. Therefore, a definitive diagnosis of GERD relies on more objective evidence of esophageal reflux. Endoscopic examination can reveal GERD complications such as erosive esophagitis and also helps in ruling out other diseases like tumors. However, in assessing the morphology of the esophagogastric junction (EGJ), endoscopic descriptions are often limited to the presence of hiatal hernia (i.e., Hill Grade IV) and lack accuracy and repeatability, especially in minor lesions [28, 29]. Furthermore, even in the absence of a hiatal hernia, the mere disappearance of GEFV (i.e., Hill Grade III) may also be associated with pathological GER and erosive esophagitis. This indicates that for patients exhibiting GERD symptoms, relying solely on the presence or absence of a hiatal hernia in endoscopic reports is insufficient; a detailed assessment of Hill classification is also necessary. While Hill classification has high consistency in clinical observations and numerous advantages, information about GEFV is rarely routinely recorded in endoscopic reports. Therefore, refining the assessment of Hill classification is of significant importance for more accurate diagnosis and understanding of GERD.

The application of deep learning in the field of gastrointestinal endoscopy has demonstrated its immense potential in gastroenterological endoscopic diagnosis. In this study, we utilized the advanced deep learning model EfficientNet-Hill for the automated Hill classification of gastroesophageal flap valve (GEFV) images. On a external test set comprising 219 GEFV images, the model achieved an accuracy of 0.833, surpassing the 0.758 accuracy of junior endoscopists and closely approaching the 0.865 accuracy of senior endoscopists. More importantly, in terms of image processing speed, the model required only 8.6 seconds to complete the recognition task for the entire external test set, showing a significant speed advantage compared to endoscopists. The use of the ONNX format enhanced the model’s interoperability across deep learning frameworks, enabling successful deployment on various computing platforms, including local computers and web frontends. While achieving processing speeds of over 50 frames per second (fps), the model ensured real-time and accurate lesion classification. This efficient automatic classification approach, which does not require additional hardware investments, provides technical support to resource-limited endoscopy centers, enhancing the speed and accuracy of medical image analysis. The future clinical application of this AI model paves the way for routine detailed Hill classification of GEFV in gastroscopy reports.

In this study, we found that CNN models significantly outperformed Transformer models in the task of automated Hill classification. Despite the excellent performance of Transformer models in many NLP and image processing tasks [30, 31], traditional CNN structures like EfficientNet surpassed the best Transformer models in key performance metrics such as Accuracy, Precision, Recall, and F1-Score. This outcome can be attributed to the advantages of CNN models, particularly EfficientNet, in capturing local structures and spatial hierarchies in images [32, 33], which is especially critical in GEFV image classification. On the other hand, Transformer models, especially the Vision Transformer (ViT), typically require large datasets to unlock their potential [34]. With smaller datasets, EfficientNet might more effectively prevent overfitting, hence showing better performance on the test set. In the future, we plan to incorporate more GEFV images to further explore the potential of Transformer architecture models.

In this study, we conducted a thorough interpretability analysis of the EfficientNet-Hill model, utilizing two techniques: Grad-CAM and SHAP. Although deep learning models are often considered inscrutable "black boxes," with the help of the torchcam library and Grad-CAM, we visualized key decision areas in the automated Hill classification model for GEFV morphologies. By analyzing the dynamic heatmaps generated by the model during the processing of GEFV endoscopic videos, we revealed the primary focus areas of the model during prediction. Additionally, using SHAP technology, we detailed the impact of each pixel on the prediction outcome. These visualization techniques not only deepened our understanding of the model’s decision-making mechanisms but also provided a basis for further improvements and optimizations of the model.

Although this study provides new insights into using deep learning for the automatic Hill grading of the gastroesophageal flap valve, several challenges remain. The dataset is limited to a single region, which may affect generalizability across diverse populations; future research will incorporate multi-center data to address this. The current gold standard relies on consensus diagnoses by endoscopists, and despite multiple expert reviewers minimizing subjectivity, inter-observer variability remains a challenge; integrating objective physiological measurements such as impedance-pH monitoring could serve as a complementary validation standard. Additionally, while Hill classification is typically performed in real time, our model was trained on static images, limiting its ability to account for dynamic factors like peristalsis and respiration. To address this, we deployed the model for real-time video inference at over 50 fps and aim to further optimize video analysis for integration into live endoscopic workflows to enhance AI-assisted decision-making.

## Conclusions

Our research employed deep learning (DL) technology to assess the function of gastroesophageal flap valves (GEFV), developing a four-category deep learning model (EfficientNet-Hill) based on the Hill classification. The study encompassed the entire process of model development, validation, testing, interpretability analysis, and multi-end deployment. On an independent external test set, EfficientNet-Hill achieved an accuracy of 83.32% and a precision of 84.81%, surpassing the performance of junior endoscopists. Moreover, the model’s inference speed significantly exceeded that of endoscopists with varying levels of experience, averaging over 50 fps. Given the importance of Hill classification in the assessment of gastroesophageal reflux disease (GERD) and its relative absence in routine endoscopic reports, our model has the potential to assist junior endoscopists in more rapidly mastering Hill classification skills and to promote its widespread application in clinical practice.

## Data Availability

The datasets analysed during the current study are available from the corresponding author on reasonable request.
